# Whole-genome sequencing and analysis of *Streptococcus iniae* strain isolated from the brain of tilapia (*Oreochromis* sp.)

**DOI:** 10.1128/mra.00493-24

**Published:** 2024-09-16

**Authors:** Anan Safwat, Garrett L. Ellward, Deborah B. Pouder, Roy P. E. Yanong, Daniel M. Czyż

**Affiliations:** 1 Microbiology and Cell Science Department, Institute of Food and Agricultural Sciences, University of Florida, Gainesville, Florida, USA; 2 Tropical Aquaculture Laboratory, School of Forest, Fisheries, & Geomatics Sciences, Institute of Food and Agricultural Sciences, University of Florida, Ruskin, Florida, USA; SUNY College of Environmental Science and Forestry, Syracuse, New York, USA

**Keywords:** *Streptococcus iniae*, fish, bacteria, pathogen, genome analysis

## Abstract

*Streptococcus iniae* is a bacterium that can infect fish, mammals, and humans. In this study, the *S. iniae*-313 strain was isolated from the brain of an infected tilapia, and the analysis of its sequenced genome is reported. The data revealed that *S. iniae*-313 carried antibiotic-resistant genes and virulence factors.

## ANNOUNCEMENT

Aquaculture continues to experience rapid growth (1). However, pathogenic bacteria pose a significant threat that can impede the growth of this agricultural commodity. One notable pathogenic bacterium is *Streptococcus iniae*, which is associated with streptococcosis in various fish species, including tilapia, eel, sturgeon, and channel catfish (2–8). The global annual financial losses attributed to *S. iniae* amount to $150 million and $10 million in the United States (9, 10).

The *S. iniae*-313 strain was isolated from the brain of an infected tilapia (*Oreochromis* sp.) at the University of Florida Tropical Aquaculture Laboratory. The bacteria were isolated by disinfecting the head after euthanasia, cutting the cranium open with a sterile scalpel, and using a sterile loop to sample and plate on tryptic soy agar (TSA) supplemented with 5% sheep’s blood, followed by a 24-h incubation at 28°C. The initial identification was made by Gram staining and metabolic profiling using the Biolog GEN III test panel.

For DNA extraction, the *S. iniae* DC-313 strain was streaked using aseptic techniques and grown at 30°C for 24 h on TSA supplemented with 5% sheep’s blood. A single colony was picked and inoculated into 10 mL of tryptic soy broth (TSB) followed by an overnight incubation at 30°C and collection of cells by centrifugation. The genomic DNA of *S. iniae*-313 was extracted using an AllPrep Bacterial DNA/RNA/Protein kit (Qiagen, USA), and short-read sequencing libraries were prepared using the tagmentation-based and PCR-based Illumina DNA Prep Kit and custom IDT 10 bp unique dual indices with a target insert size of 320 bp. After library preparation, the sequencing was performed using an Illumina NovaSeq 6000 sequencer to produce 2 × 151 bp paired-end reads for a total number of 1,880,229. Adaptor trimming and quality control were performed with BCL-Convert (version 4.1.5). After sequencing, the analysis of *S. iniae*-313 reads was completed using the Bacterial and Viral Bioinformatics Resource Center (BV-BRC) (11). Default parameters were used except where otherwise noted. The quality of the produced paired-end reads was evaluated using FastQC and trimmed using Trim Galore (12).

The *S. iniae*-313 reads were assembled using the Unicycler tool (version 0.4.8), revealing 105 contigs (13). While the length of the largest contig was ~145 kb, the contig *N*_50_ read length was 41,906 bp, and the number of contigs that construct half of the genome size (contig *L*_50_) was 16. The genome length was 2,003,645 bp with a G + C content of 36.61%.

**Fig 1:**
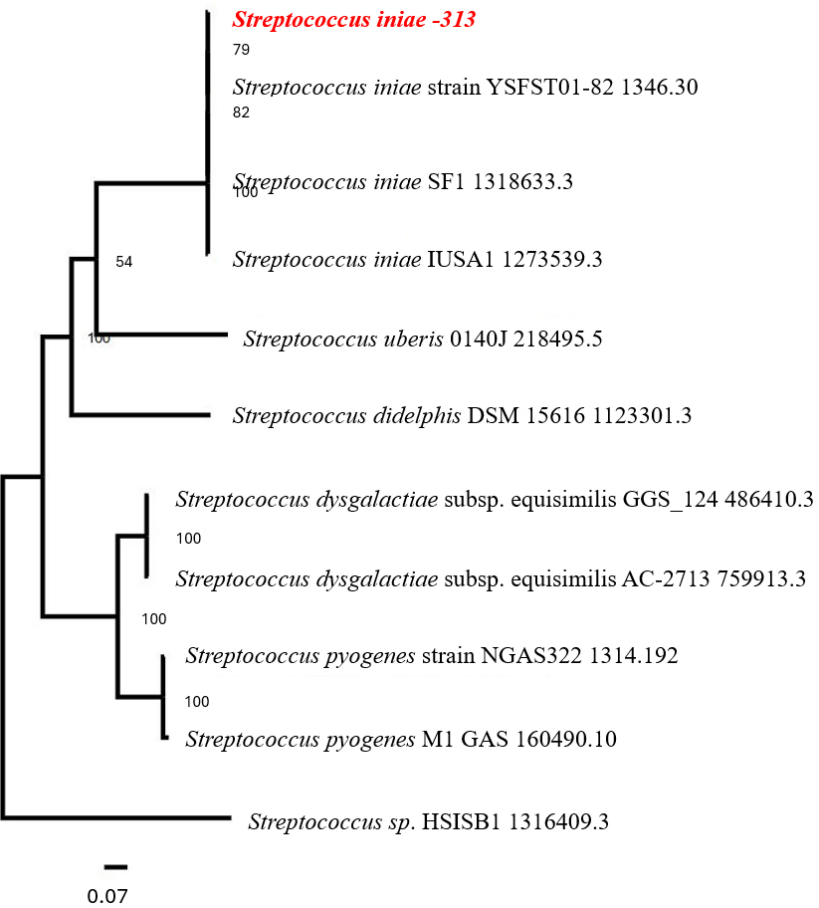
The phylogenetic tree of *S. iniae*-313 with the 10 closest genomes was generated using the phylogenetic codon tree as part of the Comprehensive Genome Analysis on BV-BRC. The genome sequences were analyzed using RAxML (20). Bootstrap values are shown at nodes. The scale bar refers to a phylogenetic distance of 0.07 nucleotide substitutions per site.

The sequence was submitted to NCBI and annotated with the Prokaryotic Genome Annotation Pipeline (PGAP 6.6) (14); however, the subsystems technology RAST tool kit (RASTtk) was used for the genome annotation described here (15). RASTtk revealed 1,983 protein-coding sequences, 44 tRNA, and 3 types of rRNA (16S, 23S, and 5S). Many of the annotated genes were found to have homology to 24 antibiotic-resistance genes (Table 1), 7 drug target genes, 20 transporter genes, and 32 virulence factor genes as assessed by PATRIC, DrugBank, TCDB, and Victors, respectively (16–19). The phylogenetic tree of *S. iniae*-313 was generated by aligning its genome with the closest 10 published genomes using the PATRIC phylogenetic codon tree (Fig. 1) (11).

**TABLE 1:** The list of the predicted AMR genes and their function

Antimicrobial resistance (AMR) mechanism	Genes
Antibiotic target in susceptible species	Alr, Ddl, EF-G, EF-Tu, folA, Dfr, folP, gyrA, gyrB, Iso-tRNA, kasA, MurA, rpoB, rpoC, S10p, S12p
Antibiotic target replacement protein	FabK
Gene conferring resistance via absence	gidB
Protein altering cell wall charge conferring antibiotic resistance	GdpD, MprF, PgsA
Regulator modulating expression of antibiotic resistance genes	LiaF, LiaR, LiaS

## Data Availability

The whole genome sequence has been deposited to Sequence Read Archive (SRA) with the accession number SRR28967390, available through the National Center for Biotechnology Information. The WGS accession number is SAMN39963917. The WGS project ID is JBAJMQ01. The RASTtk genome annotation is available at Zenodo repository DOI: 10.5281/zenodo.11210819.
